# On Interpolating Sesqui-Harmonic Maps Between Riemannian Manifolds

**DOI:** 10.1007/s12220-018-00130-x

**Published:** 2019-01-14

**Authors:** Volker Branding

**Affiliations:** Faculty of Mathematics, University of Vienna, Oskar-Morgenstern-Platz 1, 1090 Vienna, Austria

**Keywords:** Interpolating sesqui-harmonic maps, Harmonic maps, Biharmonic maps, Bosonic string with extrinsic curvature term, 58E20, 31B30

## Abstract

Motivated from the action functional for bosonic strings with extrinsic curvature term we introduce an action functional for maps between Riemannian manifolds that interpolates between the actions for harmonic and biharmonic maps. Critical points of this functional will be called interpolating sesqui-harmonic maps. In this article we initiate a rigorous mathematical treatment of this functional and study various basic aspects of its critical points.

## Introduction and Results

*Harmonic maps* play an important role in geometry, analysis and physics. On the one hand, they are one of the most studied variational problems in geometric analysis, and on the other hand they naturally appear in various branches of theoretical physics, for example as critical points of the non-linear sigma model or in the theory of elasticity. Mathematically, they are defined as critical points of the Dirichlet energy

1.1 $$\begin{aligned} E_1(\phi )=\int _M|\mathrm{{d}}\phi |^2\text {d}V, \end{aligned}$$

where $\phi :M\rightarrow N$ is a map between the two Riemannian manifolds $(M,h)$ and $(N,g)$. The critical points of (1.1) are characterized by the vanishing of the so-called *tension field*, which is given by

$$\begin{aligned} 0=\tau (\phi ):={\text {Tr}}_h\nabla \mathrm{{d}}\phi . \end{aligned}$$

This is a semilinear, elliptic second-order partial differential equation, for which many results on existence and qualitative behavior of its solutions have been obtained. For a recent survey on harmonic maps see [15]. Due to their non-linear nature, harmonic maps do not always need to exist. For example, if $M=T^2$ and $N=S^2$, there does not exist a harmonic map with $\deg \phi =\pm 1$ regardless of the chosen metrics [12].

In these cases one may consider the following generalization of the harmonic map equation, the so-called *biharmonic maps*. These arise as critical points of the bienergy [16], which is defined as

1.2 $$\begin{aligned} E_2(\phi )=\int _M|\tau (\phi )|^2\text {d}V. \end{aligned}$$

In contrast to the harmonic map equation, the biharmonic map equation is an elliptic equation of fourth order and is characterized by the vanishing of the *bitension field*

$$\begin{aligned} 0=\tau _2(\phi ):=\Delta \tau (\phi )-R^N(\mathrm{{d}}\phi (e_\alpha ),\tau (\phi ))\mathrm{{d}}\phi (e_\alpha ), \end{aligned}$$

where $\Delta$ is the connection Laplacian on $\phi ^*TN$, $e_\alpha$ an orthonormal basis of $TM$, and $R^N$ denotes the curvature tensor of the target manifold $N$. We make use of the Einstein summation convention, meaning that we sum over repeated indices.

In the literature that studies analytical aspects of biharmonic maps, one refers to (1.2) as the energy functional for *intrinsic biharmonic maps*.

For a survey on biharmonic maps between Riemannian manifolds, we refer to [10] and [26].

In this article we want to focus on the study of an action functional that interpolates between the actions for harmonic and biharmonic maps

1.3 $$\begin{aligned} E_{\delta _1,\delta _2}(\phi )=\delta _1\int _M|\mathrm{{d}}\phi |^2\text {d}V+\delta _2\int _M|\tau (\phi )|^2\text {d}V\end{aligned}$$

with $$\delta _1, \delta _2\in \mathbb {R}$$.

This functional appears at several places in the physics literature. In string theory it is known as *bosonic string with extrinsic curvature term*, see [17, 28].

On the mathematical side there have been several articles dealing with some particular aspect of (1.3). Up to the best knowledge of the author the first place where the functional (1.3) was mentioned is [13, pp.134–135] with $\delta _2=1$ and $\delta _1>0$. In that reference it is already shown that if the domain has dimension 2 or 3 and the target $N$ negative sectional curvature then the critical points of (1.3) reduce to harmonic maps. Later it was shown in [20, p.191] that no critical points exist if one does not impose the curvature condition on $N$ and also assumes that $\deg \phi =1$. Some analytic questions related to critical points of (1.3) have been discussed in [19] assuming $\delta _1=2,\delta _2=1$. For the sake of completeness we want to mention that the functional (1.3) with $\delta _1>0$ and $\delta _2=\frac{1}{2}$ is also presented in the survey article “A report on harmonic maps”, see [11, p.28, Example (6.30)].

In [21] the authors initiate an extensive study of (1.3) assuming $\delta _2=1$ and $\delta _1\in \mathbb {R}$ under the condition that $\phi$ is an immersion. They consider variations of (1.3) that are normal to the image $\phi (M)\subset N$. In this setup they call critical points of (1.3) *biminimal immersions*. They also point out possible applications of their model to the theory of elasticity.

Up to now there exist several results on biminimal immersions, see for example [9] for biminimal hypersurfaces into spheres, [23] for biminimal submanifolds in manifolds of non-positive curvature and [24] for biminimal submanifolds of Euclidean space. Instead of investigating maps that are immersions, we here want to put the focus on arbitrary maps between Riemannian manifolds.

The critical points of (1.3) will be referred to as *interpolating sesqui-harmonic maps* and are given by

1.4 $$\begin{aligned} \delta _2\Delta \tau (\phi )=\delta _2 R^N(\mathrm{{d}}\phi (e_\alpha ),\tau (\phi ))\mathrm{{d}}\phi (e_\alpha )+\delta _1\tau (\phi ), \end{aligned}$$

where $\tau (\phi )$ denotes the tension field of the map $\phi$ and by $\Delta$ we are representing the connection Laplacian on the vector bundle $\phi ^*TN$.

As in the case of biharmonic maps, it is obvious that harmonic maps solve (1.4). For this reason we are mostly interested in solutions of (1.4) that are not harmonic maps. However, we can expect that as in the case of biharmonic maps there may be many situations in which solutions of (1.4) will be harmonic maps. In particular, we can expect that this is the case if $N$ has negative sectional curvature and $\delta _1\delta _2>0$. This question will be dealt with in Sect. 4. On the other hand, if $\delta _1$ and $\delta _2$ have opposite sign, we might expect a different behavior of solutions of (1.4) since in this case the two terms in the energy functional (1.3) are competing with each other and the energy functional can become unbounded from above and below.

This article is organized as follows: In Sect. 2 we study basic features of interpolating sesqui-harmonic maps. Afterwards, in Sect. 3, we derive several explicit solutions of the interpolating sesqui-harmonic map equation and in the last section we provide several results that characterize the qualitative behavior of interpolating sesqui-harmonic maps.

Throughout this paper we will make use of the following conventions. Whenever choosing local coordinates we will use Greek letters to denote indices on the domain $M$ and Latin letters for indices on the target $N$. We will choose the following convention for the curvature tensor $R(X,Y)Z:=[\nabla _X,\nabla _Y]Z-\nabla _{[X,Y]}Z$ such that the sectional curvature is given by $K(X,Y)=R(X,Y,Y,X)$. For the Laplacian acting on functions $f\in C^\infty (M)$ we choose the convention $\Delta f={\text {div}}\,{\text {grad}}\,f$, and for sections in the vector bundle $\phi ^{*}TN$ we make the choice $\Delta ^{\phi ^*TN}={\text {Tr}}(\nabla ^{\phi ^*TN}\nabla ^{\phi ^*TN})$. Note that the connection Laplacian on $\phi ^*TN$ is defined by $\Delta :=\nabla _{e_\alpha }\nabla _{e_\alpha }-\nabla _{\nabla _{e_\alpha }e_\alpha }$.

## Interpolating Sesqui-Harmonic Maps

In this section we analyze the basic features of the action functional (1.3) and start by calculating its critical points.

### Proposition 2.1

The critical points of (1.3) are given by

$$\begin{aligned} \delta _2\Delta \tau (\phi )=\delta _2 R^N(\mathrm{{d}}\phi (e_\alpha ),\tau (\phi ))\mathrm{{d}}\phi (e_\alpha )+\delta _1\tau (\phi ), \end{aligned}$$

where $\tau (\phi ):=\mathrm{Tr}_{\mathrm{h}}\nabla \mathrm{d}\phi$ is the tension field of the map $\phi$.

### Proof

We choose Riemannian normal coordinates that satisfy $\nabla _{\partial _t}e_\alpha =0$ at the respective point. Consider a variation of the map $\phi$, that is $\phi _t:(-\varepsilon ,\varepsilon )\times M\rightarrow N$, which satisfies $\frac{\nabla }{\partial t}\phi \big |_{t=0}=\eta$. It is well known that

$$\begin{aligned} \frac{\mathrm{{d}}}{\mathrm{{d}}t}\big |_{t=0}\int _M|\mathrm{{d}}\phi _t|^2\text {d}V=-2\int _M\langle \eta ,\tau (\phi )\rangle \text {d}V. \end{aligned}$$

In addition, we find

$$\begin{aligned} \frac{\mathrm{{d}}}{\mathrm{{d}}t}\big |_{t=0}\int _M|\tau (\phi _t)|^2\text {d}V=&2\int _M \langle \frac{\nabla }{\partial t}\nabla _{e_\alpha }\mathrm{{d}}\phi _t(e_\alpha ),\tau (\phi _t)\rangle \text {d}V\big |_{t=0}\\ =&2\int _M\big (\langle R^N(\mathrm{{d}}\phi _t(\partial _t),\mathrm{{d}}\phi _t(e_\alpha ))\mathrm{{d}}\phi _t(e_\alpha ),\tau (\phi _t)\rangle \\&+\langle \nabla _{e_\alpha }\nabla _{e_\alpha }\mathrm{{d}}\phi _t(\partial _t),\tau (\phi _t) \rangle \big )\text {d}V\big |_{t=0}\\ =&2\int _M\langle \eta ,\Delta \tau (\phi )-R^N(\mathrm{{d}}\phi (e_\alpha ),\tau (\phi ))d \phi (e_\alpha )\rangle \text {d}V. \end{aligned}$$

Adding up both contributions yields the claim. $\square$

Solutions of (1.4) will be called *interpolating sesqui-harmonic maps*.^1^

### Remark 2.2

Choosing $\delta _1=2,\delta _2=1$ and $M=S^4,N=S^k$ solutions of (1.4) were called *quasi-biharmonic maps* in [30]. These arise when considering a sequence of weakly intrinsic biharmonic maps in dimension four. When taking the limit, one finds that quasi-biharmonic spheres separate at finitely many points as in many conformally invariant variational problems.

### Remark 2.3

There is another way how we can think of (1.4). If we interpret the biharmonic map equation as acting with the Jacobi-field operator $J$ on the tension field $\tau (\phi )$, then we may rewrite the equation for interpolating sesqui-harmonic maps as

$$\begin{aligned} J(\tau (\phi ))=\frac{\delta _1}{\delta _2}\tau (\phi ). \end{aligned}$$

Since the Jacobi-field operator is elliptic it has a discrete spectrum whenever $M$ is a closed manifold. In this case the equation for interpolating sesqui-harmonic maps can be thought of as an eigenvalue equation for the Jacobi-field operator.

### Remark 2.4

In order to highlight the dependence of the action functional (1.3) on the metric on the domain $M$, we write

$$\begin{aligned} E_{\delta _1,\delta _2}(\phi ,h)=\delta _1\int _M|\mathrm{{d}}\phi |_h^2 \text {d}V_{h}+\delta _2\int _M|\tau _h(\phi )|_h^2\text {d}V_{h}, \end{aligned}$$

where $\text {d}V_h$ represents the volume element of the metric $h$. If we perform a rescaling of the metric by a constant factor $\tilde{h}:=\lambda ^2h$, the action functional transforms as

$$\begin{aligned} E_{\delta _1,\delta _2}(\phi ,\tilde{h})=\delta _1\int _M|\mathrm{{d}}\phi |_h^2\lambda ^{n-2}\text {d}V_h + \delta _2\int _M|\tau _h(\phi )|_h^2\lambda ^{n-4}\text {d}V_h. \end{aligned}$$

This clearly reflects the fact that the action functional for harmonic maps is scale-invariant in two dimensions, whereas the action functional for biharmonic maps is scale-invariant in four dimensions. We can conclude that the action for interpolating sesqui-harmonic maps is not scale-invariant in any dimension and we may expect that interpolating sesqui-harmonic maps may be most interesting if $\dim M=2,3,4$.

In order to highlight the analytical structure of (1.4) we take a look at the case of a spherical target. For biharmonic maps this was carried out in [18] making use of a different method.

### Proposition 2.5

For $\phi :M\rightarrow S^n\subset \mathbb {R}^{n+1}$ with the constant curvature metric, the equation for interpolating sesqui-harmonic maps (1.4) acquires the form

2.1 $$\begin{aligned}&\delta _2(\Delta ^2\phi +(|\Delta \phi |^2+\Delta |\mathrm{{d}}\phi |^2+2\langle \mathrm{{d}}\phi ,\nabla \Delta \phi \rangle +2|\mathrm{{d}}\phi |^4)\phi +2\nabla (|\mathrm{{d}}\phi |^2\mathrm{d}\phi ))\nonumber \\&\quad =\delta _1(\Delta \phi +|\mathrm{{d}}\phi |^2\phi ). \end{aligned}$$

### Proof

Recall that for a spherical target the tension field has the simple form

$$\begin{aligned} \tau (\phi )=\Delta \phi +|\mathrm{{d}}\phi |^2\phi . \end{aligned}$$

Since we assume that $N=S^n$ with constant curvature, the term on the right-hand side of (1.4) acquires the form

$$\begin{aligned} -R^N(\mathrm{{d}}\phi (e_\alpha ),\tau (\phi ))\mathrm{{d}}\phi (e_\alpha )=&|\mathrm{{d}}\phi |^2\tau (\phi )-\langle \mathrm{{d}}\phi (e_\alpha ),\tau (\phi )\rangle \mathrm{{d}}\phi (e_\alpha )\\ =&|\mathrm{{d}}\phi |^2\Delta \phi +|\mathrm{{d}}\phi |^4\phi -\langle \mathrm{{d}}\phi (e_\alpha ),\Delta \phi \rangle \mathrm{{d}}\phi (e_\alpha ). \end{aligned}$$

Using the special structure of the Levi-Civita connection on $S^n\subset \mathbb {R}^{n+1}$, we calculate

$$\begin{aligned} \Delta \tau (\phi )=&\nabla \nabla (\Delta \phi +|\mathrm{{d}}\phi |^2\phi ) \\ =&\nabla (\nabla \Delta \phi +\langle \mathrm{{d}}\phi ,\Delta \phi \rangle \phi +\nabla (|\mathrm{{d}}\phi |^2\phi ))\\ =&\Delta ^2\phi +\langle \mathrm{{d}}\phi ,\nabla \Delta \phi \rangle \phi +\nabla (\langle \mathrm{{d}}\phi ,\Delta \phi \rangle \phi ) +\Delta (|\mathrm{{d}}\phi |^2\phi )+\langle \mathrm{{d}}\phi ,\nabla (|\mathrm{{d}}\phi |^2\phi )\rangle \phi \\ =&\Delta ^2\phi +|\Delta \phi |^2\phi +2\langle \mathrm{{d}}\phi ,\nabla \Delta \phi \rangle \phi +\langle \mathrm{{d}}\phi ,\Delta \phi \rangle \mathrm{{d}}\phi +\Delta (|\mathrm{{d}}\phi |^2\phi )+|\mathrm{{d}}\phi |^4\phi . \end{aligned}$$

Combining both equations yields the claim. $\square$

By varying (1.3) with respect to the domain metric, we obtain the energy-momentum tensor. Since the energy-momentum tensor for both harmonic and biharmonic maps is well known in the literature, we can directly give the desired result.

### Proposition 2.6

The energy-momentum tensor associated to (1.3) is given by

2.2 $$\begin{aligned} T(X,Y)&=\delta _1(\langle \mathrm{{d}}\phi (X),\mathrm{d}\phi (Y)\rangle -\frac{1}{2}|\mathrm{{d}}\phi |^2h(X,Y)) \nonumber \\&\quad +\delta _2\big (\frac{1}{2}|\tau (\phi )|^2h(X,Y)+\langle \mathrm{{d}}\phi ,\nabla \tau (\phi )\rangle h(X,Y) \nonumber \\&\quad -\langle \mathrm{{d}}\phi (X),\nabla _Y\tau (\phi )\rangle -\langle \mathrm{{d}}\phi (Y),\nabla _X\tau (\phi )\rangle \big ), \end{aligned}$$

where $X,Y$ are vector fields on $M$.

### Proof

We consider a variation of the metric on $M$, that is

$$\begin{aligned} \frac{\mathrm{{d}}}{\mathrm{{d}}t}\big |_{t=0}h_t=k, \end{aligned}$$

where $k$ is a symmetric $(2,0)$-tensor. The energy-momentum tensor for harmonic maps can be computed as [2]

$$\begin{aligned} \frac{\mathrm{{d}}}{\mathrm{{d}}t}\big |_{t=0}\int _M|\mathrm{{d}}\phi |^2\text {d}V_{h_t} =\int _M k^{\alpha \beta }\big (\langle \mathrm{{d}}\phi (e_\alpha ),\mathrm{{d}}\phi (e_\beta )\rangle -\frac{1}{2}|\mathrm{{d}}\phi |^2h_{\alpha \beta }\big )\text {d}V_h. \end{aligned}$$

Deriving the energy-momentum tensor for biharmonic maps is more involved as one has to vary the connection of the domain since it depends on the metric. The energy-momentum tensor for biharmonic maps was already presented in [16], a rigorous derivation was obtained in [22, Theorem 2.4], that is

$$\begin{aligned} \frac{\mathrm{{d}}}{\mathrm{{d}}t}\big |_{t=0}\int _M|\tau (\phi )|^2\text {d}V_{h_t}&=-\int _M k^{\alpha \beta }(\frac{1}{2}|\tau (\phi )|^2h_{\alpha \beta }+\langle \mathrm{{d}}\phi ,\nabla \tau (\phi )\rangle h_{\alpha \beta } \\&\quad -\langle \mathrm{{d}}\phi (e_\alpha ),\nabla _{e_\beta }\tau (\phi )\rangle -\langle \mathrm{{d}}\phi (e_\beta ),\nabla _{e_\alpha }\tau (\phi )\rangle )\text {d}V_h. \end{aligned}$$

Combining both formulas concludes the proof. $\square$

It can be directly seen that the energy-momentum tensor (2.2) is symmetric. For the sake of completeness we prove the following:

### Proposition 2.7

The energy-momentum tensor (2.2) is divergence free.

### Proof

We choose a local orthonormal basis $e_\alpha$ and set

$$\begin{aligned} T_{\alpha \beta }:=T(e_\alpha ,e_\beta )=&\delta _1\left( \langle \mathrm{{d}}\phi (e_\alpha ),\mathrm{{d}}\phi (e_\beta )\rangle -\frac{1}{2}|\mathrm{{d}}\phi |^2h_{\alpha \beta }\right) \\&+\delta _2\big (\frac{1}{2}|\tau (\phi )|^2h_{\alpha \beta }+\langle \mathrm{{d}}\phi ,\nabla \tau (\phi )\rangle h_{\alpha \beta } \nonumber \\&-\langle \mathrm{{d}}\phi (e_\alpha ),\nabla _{e_\beta }\tau (\phi )\rangle -\langle \mathrm{{d}}\phi (e_\beta ),\nabla _{e_\alpha }\tau (\phi )\rangle \big ).\nonumber \end{aligned}$$

By a direct calculation we find

$$\begin{aligned} \nabla _{e_\alpha }\left( \langle \mathrm{{d}}\phi (e_\alpha ),\mathrm{{d}}\phi (e_\beta )\rangle -\frac{1}{2}|\mathrm{{d}}\phi |^2h_{\alpha \beta }\right) =\langle \tau (\phi ),\mathrm{{d}}\phi (e_\beta )\rangle \end{aligned}$$

and also

$$\begin{aligned} \nabla _{e_\alpha }&(\frac{1}{2}|\tau (\phi )|^2h_{\alpha \beta }+\langle \mathrm{{d}}\phi ,\nabla \tau (\phi )\rangle h_{\alpha \beta } -\langle \mathrm{{d}}\phi (e_\alpha ),\nabla _{e_\beta }\tau (\phi )\rangle -\langle \mathrm{{d}}\phi (e_\beta ),\nabla _{e_\alpha }\tau (\phi )\rangle ) \\ =&\langle \nabla _{e_\beta }\tau (\phi ),\tau (\phi )\rangle +\langle \nabla _{e_\beta } \mathrm{{d}}\phi (e_\gamma ),\nabla _{e_\gamma }\tau (\phi )\rangle +\langle \mathrm{{d}}\phi (e_\gamma ),\nabla _{e_\beta }\nabla _{e_\gamma }\tau (\phi )\rangle \\&-\langle \tau (\phi ),\nabla _{e_\beta }\tau (\phi )\rangle -\langle \mathrm{{d}}\phi (e_\alpha ),\nabla _{e_\alpha }\nabla _{e_\beta }\tau (\phi )\rangle \\&-\langle \nabla _{e_\alpha }\mathrm{{d}}\phi (e_\beta ),\nabla _{e_\alpha }\tau (\phi )\rangle -\langle \mathrm{{d}}\phi (e_\beta ),\nabla \nabla \tau (\phi )\rangle \\ =&\langle \mathrm{{d}}\phi (e_\alpha ),R^N(\mathrm{{d}}\phi (e_\beta ),\mathrm{{d}}\phi (e_\alpha ))\tau (\phi )\rangle -\langle \mathrm{{d}}\phi (e_\beta ),\Delta \tau (\phi )\rangle , \end{aligned}$$

where we used the torsion-freeness of the Levi-Civita connection. Adding up both equations yields the claim. $\square$

### Conservation Laws for Targets with Symmetries

In this subsection we discuss how to obtain a conservation law for solutions of the interpolating sesqui-harmonic map equation in the case that the target manifold has a certain amount of symmetry, more precisely, if it possesses Killing vector fields. A similar discussion has been performed in [4].

To this end let $\xi$ be a diffeomorphism that generates a one-parameter family of vector fields $X$. Then we know that

$$\begin{aligned} \frac{\mathrm{{d}}}{\mathrm{{d}}t}\big |_{t=0}\xi ^*g=\mathcal {L}_Xg, \end{aligned}$$

where $\mathcal {L}$ denotes the Lie-derivative acting on the metric. In terms of local coordinates the Lie-derivative of the metric is given by

$$\begin{aligned} \mathcal {L}_Xg_{ij}=\nabla _iX_j+\nabla _jX_i. \end{aligned}$$

This enables us to give the following:

#### Definition 2.8

Let $\xi$ be a diffeomorphism that generates a one-parameter family of vector fields $X$ on $N$. We say that $X$ generates a symmetry for the action $E_{\delta _1,\delta _2} (\phi ,\xi ^*g)$ if

$$\begin{aligned} \frac{\mathrm{{d}}}{\mathrm{{d}}t}\big |_{t=0}E_{\delta _1,\delta _2}(\phi ,\xi ^*g)=\int _M\mathcal {L}_X(\delta _1|\mathrm{{d}}\phi |^2+\delta _2|\tau (\phi )|^2)\text {d}V=0, \end{aligned}$$

where the Lie-derivative is acting on the metric $g$.

Note that if $X$ generates an isometry then $\mathcal {L}_Xg=0$ such that we have to require the existence of Killing vector fields on the target.

In the following we will make use of the following facts:

#### Lemma 2.9

If $X$ is a Killing vector field on the target $N$, then

2.3 $$\begin{aligned} \nabla ^2_{Y,Z}X&=-R^N(X,Y)Z, \end{aligned}$$

2.4 $$\begin{aligned} \mathcal {L}_X\Gamma ^k_{ij}&=\nabla _i\nabla _jX^k-R^k_{~{ijl}}X^l, \end{aligned}$$

where $$\Gamma ^k_{ij}$$ are the Christoffel symbols on N, $$ R^k_{~ijl}$$ the components of the Riemannian curvature tensor on N and $$Y,Z$$ vector fields on $$N$$.

#### Lemma 2.10

Let $\phi :M\rightarrow N$ be a smooth solution of (1.4) and assume that $N$ admits a Killing vector field $X$. Then the Lie-derivative acting on the metric $g$ of the energy density is given by

$$\begin{aligned} \mathcal {L}_X(\delta _1|\mathrm{{d}}\phi |^2+\delta _2|\tau (\phi )|^2) =&2\delta _1\nabla _{e_\alpha }\langle \mathrm{{d}}\phi (e_\alpha ),X(\phi )\rangle \nonumber \\&+2\delta _2\nabla _{e_\alpha }(\langle \tau (\phi ),\nabla _{e_\alpha }X(\phi )\rangle -\delta _2\langle \nabla _{e_\alpha }\tau (\phi ),X(\phi )\rangle ). \end{aligned}$$

#### Proof

We choose Riemannian normal coordinates $x_\alpha$ on $M$ and calculate

$$\begin{aligned} \mathcal {L}_X|\mathrm{{d}}\phi |^2=&\,(\mathcal {L}_Xg)_{ij}\frac{\partial \phi ^i}{\partial x^\alpha } \frac{\partial \phi ^j}{\partial x^\beta }h^{\alpha \beta } \\ =\,&2\nabla _iX_j\frac{\partial \phi ^i}{\partial x^\alpha }\frac{\partial \phi ^j}{\partial x^\beta }h^{\alpha \beta } \\ =\,&2\langle \mathrm{{d}}\phi (e_\alpha ),\nabla _{e_\alpha }(X(\phi ))\rangle \\ =\,&2\nabla _{e_\alpha }\langle \mathrm{{d}}\phi (e_\alpha ),X(\phi )\rangle -2\langle \tau (\phi ),X(\phi )\rangle . \end{aligned}$$

To calculate the variation of the tension field with respect to the target metric, we first of all note that

$$\begin{aligned} \mathcal {L}_X|\tau (\phi )|^2=\,&2\tau ^i(\phi )\tau ^j(\phi )\nabla _iX_j+2g_{ij}\tau ^i(\phi ) \mathcal {L}_X\tau ^j(\phi ). \end{aligned}$$

Making use of the local expression of the tension field this yields

$$\begin{aligned} \mathcal {L}_X\tau ^j(\phi )=\,&\mathcal {L}_X\left( \Delta \phi ^j+h^{\alpha \beta }\frac{\partial \phi ^k}{\partial x^\alpha }\frac{\partial \phi ^l}{\partial x^\beta }\Gamma ^j_{kl}\right) \\ =\,&h^{\alpha \beta }\frac{\partial \phi ^k}{\partial x^\alpha }\frac{\partial \phi ^l}{\partial x^\beta }\mathcal {L}_X\Gamma ^j_{kl} \\ =\,&h^{\alpha \beta }\frac{\partial \phi ^k}{\partial x^\alpha }\frac{\partial \phi ^l}{\partial x^\beta }(\nabla _k\nabla _lX^j-R^j_{~klr}X^r), \end{aligned}$$

where we used (2.4) in the last step.

This allows us to infer

$$\begin{aligned} g_{ij}\tau ^i(\phi )\mathcal {L}_X\tau ^j(\phi )=\langle \tau (\phi ),\nabla _{\mathrm{{d}}\phi (e_\alpha )} \nabla _{e_\alpha }X(\phi )\rangle +\langle R^N(\tau (\phi ),\mathrm{{d}}\phi (e_\alpha ))\mathrm{{d}}\phi (e_\alpha ),X\rangle . \end{aligned}$$

Combining both equations we find

$$\begin{aligned} \mathcal {L}_X|\tau (\phi )|^2=\,&2\nabla _{e_\alpha }\langle \tau (\phi ),\nabla _{e_\alpha } X(\phi )\rangle -2\nabla _{e_\alpha }\langle \nabla _{e_\alpha }\tau (\phi ),X(\phi )\rangle +2\langle \Delta \tau (\phi ),X(\phi )\rangle \\&+2\langle R^N(\tau (\phi ),\mathrm{{d}}\phi (e_\alpha ))\mathrm{{d}}\phi (e_\alpha ),X\rangle . \end{aligned}$$

The result follows by adding up both contributions. $\square$

#### Proposition 2.11

Let $\phi :M\rightarrow N$ be a smooth solution of (1.4) and assume that $N$ admits a Killing vector field $X$. Then the following vector field is divergence free

2.5 $$\begin{aligned} J_\alpha :=\delta _1\langle \mathrm{{d}}\phi (e_\alpha ),X(\phi )\rangle +\delta _2\langle \tau (\phi ),\nabla _{e_\alpha }X(\phi )\rangle -\delta _2\langle \nabla _{e_\alpha }\tau (\phi ),X(\phi )\rangle . \end{aligned}$$

#### Proof

A direct calculation yields

$$\begin{aligned} \nabla _{e_\alpha }J_\alpha =\,&\delta _1\langle \tau (\phi ),X(\phi )\rangle +\delta _1\underbrace{\langle \mathrm{{d}}\phi (e_\alpha ),\nabla _{e_\alpha } X(\phi )\rangle }_{=0} +\delta _2\langle \tau (\phi ),\nabla ^2_{e_\alpha ,e_\alpha } X(\phi )\rangle \\&-\delta _2\langle \Delta \tau (\phi ),X(\phi )\rangle \\ =\,&\langle X(\phi ),\delta _1\tau (\phi )+\delta _2R^N(\mathrm{{d}}\phi (e_\alpha ),\tau (\phi )) \mathrm{{d}}\phi (e_\alpha )-\delta _2\Delta \tau (\phi )\rangle \\ =\,&0, \end{aligned}$$

where we used (2.3) and that $\phi$ is a solution of (1.4) in the last step. $\square$

#### Remark 2.12

In the physics literature the vector field (2.5) is usually called *Noether current*.

## Explicit Solutions of the Interpolating Sesqui-Harmonic Map Equation

In this section we want to derive several explicit solutions to the Euler–Lagrange equation (1.4). We can confirm that solutions may have a different behavior than biharmonic or harmonic maps.

Let us start in the most simple setup possible.

### Example 3.1

Suppose that $M=N=S^1$ and by $s$ we denote the global coordinate on $S^1$. Then (1.4) acquires the form

$$\begin{aligned} \delta _2\phi ^{''''}(s)=\delta _1\phi ^{''}(s). \end{aligned}$$

Taking an ansatz of the form

$$\begin{aligned} \phi (s)=\sum _k a_ke^{iks} \end{aligned}$$

we obtain

$$\begin{aligned} \sum _k a_kk^2(\delta _2k^2-\delta _1)e^{iks}=0. \end{aligned}$$

Consequently, we have to impose the condition $k^2=\frac{\delta _1}{\delta _2}$. In particular, this shows that there does not exist a solution of (1.4) on $S^1$ if $\delta _1$ and $\delta _2$ have opposite sign.

### Example 3.2

Consider the case $M=\mathbb {R}^2$ and $N=\mathbb {R}$. In this case being interpolating sesqui-harmonic means to find a function $f:M\rightarrow N$ that solves

$$\begin{aligned} \delta _2(\partial ^2_x+\partial ^2_y)^2f=\delta _1(\partial ^2_x+\partial ^2_y)f, \end{aligned}$$

where $x,y$ denote the canonical coordinates in $\mathbb {R}^2$. If we make a separation ansatz of the form

$$\begin{aligned} f(x,y)=e^{\alpha x}e^{\beta y}, \end{aligned}$$

then we are lead to the following algebraic expression

$$\begin{aligned} \delta _2(\alpha ^2+\beta ^2)=\delta _1. \end{aligned}$$

Let us distinguish the following cases:

1. $\delta _1=0$, that is $f$ is biharmonic. In this case $\alpha ^2+\beta ^2=0$ and we have to choose $\alpha ,\beta \in {\mathbb {C}}$.
2. $\delta _2=0$, that is $f$ is harmonic. In this case there are no restrictions on $\alpha ,\beta$.
3. If $\delta _1\delta _2>0$ we have to impose the condition $\alpha ^2+\beta ^2>0$ meaning that $\alpha ,\beta \in \mathbb {R}$.
4. If $\delta _1\delta _2<0$ we find that $\alpha ^2+\beta ^2<0$ meaning that $\alpha ,\beta \in {\mathbb {C}}$.

This again shows that interpolating sesqui-harmonic functions may be very different from both harmonic and biharmonic functions.

### Interpolating Sesqui-Harmonic Functions in Flat Space

In this section we study interpolating sesqui-harmonic functions in flat space.

First, suppose that $M=N=\mathbb {R}$ and we denote the global coordinate on $\mathbb {R}$ by $x$. Then (1.4) acquires the form

$$\begin{aligned} \delta _2\phi ^{''''}(x)=\delta _1\phi ^{''}(x). \end{aligned}$$

This can be integrated as

$$\begin{aligned} \phi (x)=\frac{\delta _2}{\delta _1}\left( c_1e^{\sqrt{\frac{\delta _1}{\delta _2}}x} +c_2e^{-\sqrt{\frac{\delta _1}{\delta _2}}x}\right) +c_3x+c_4, \end{aligned}$$

where $c_i,i=1,\ldots 4$ are integration constants. It is interesting to note that both limits $\delta _1\rightarrow 0$ and $\delta _2\rightarrow 0$ do not exist. Consequently, the solution of the interpolating sesqui-harmonic function equation does neither reduce to a solution of the harmonic or the biharmonic function equation. In the following we will analyze if the same behavior persists in higher dimensions.

If we take $M=(\mathbb {R}^n,\delta )$ and $N=(\mathbb {R},\delta )$ both with the Euclidean metric, the equation for interpolating sesqui-harmonic maps turns into

3.1 $$\begin{aligned} \delta _2\Delta \Delta f=\delta _1\Delta f, \end{aligned}$$

where $f:\mathbb {R}^n\rightarrow \mathbb {R}$. Although this equation is linear, we may expect some analytical difficulties since we do not have a maximum principle available for fourth-order equations.

A full in detail analysis of this equation is far beyond the scope of this article. Nevertheless, we will again see that solutions of (3.1) may be very different from harmonic and biharmonic functions. We will be looking for radial solutions of (3.1), where $r:=\sqrt{x_1^2+\ldots +x_n^2}$. In this case the Laplacian has the form

$$\begin{aligned} \Delta =\frac{\mathrm{{d}}^2}{\mathrm{{d}}r^2}+\frac{n-1}{r}\frac{\mathrm{{d}}}{\mathrm{{d}}r}. \end{aligned}$$

Recall that the fundamental solution of the Laplace equation in $\mathbb {R}^n$ is given by

$$\begin{aligned} H_{\Delta }(x,y)= {\left\{ \begin{array}{ll} |x-y|^{2-n},&{} n\ge 3, \\ \log |x-y|,&{} n=2, \end{array}\right. } \end{aligned}$$

whereas for biharmonic functions it acquires the form

$$\begin{aligned} H_{\Delta ^2}(x,y)= {\left\{ \begin{array}{ll} |x-y|^{4-n},&{} n\ge 5, \\ \log |x-y|,&{} n=4,\\ |x-y|,&{} n=3. \end{array}\right. } \end{aligned}$$

Note that we did not write down any normalization of the fundamental solutions.

We cannot expect to find a unique solution to (3.1) since we can always add a harmonic function once we have constructed a solution to (3.1). Since we are considering $\mathbb {R}^n$ instead of a curved manifold at the moment, all curvature terms in (1.4) vanish and we are dealing with a linear problem.

Instead of trying to directly solve (3.1) we rewrite the equation as follows:

$$\begin{aligned} \Delta \left( \Delta f-\frac{\delta _1}{\delta _2}f\right) =0. \end{aligned}$$

Assume that $n\ge 3$ and making use of the fundamental solution of the Laplacian, we may solve

$$\begin{aligned} \Delta f(r)-\frac{\delta _1}{\delta _2}f(r)=r^{2-n}, \end{aligned}$$

which then provides an interpolating sesqui-harmonic function. This yields the following ordinary differential equation

3.2 $$\begin{aligned} f''(r)+\frac{n-1}{r}f'(r)-\frac{\delta _1}{\delta _2}f(r)=r^{2-n}. \end{aligned}$$

This equation can be solved explicitly in terms of a linear combination of Bessel functions in any dimension. Since the general solution is rather lengthy, we only give some explicit solutions for a fixed dimension.

- Suppose that $n=3$, then the solution of (3.2) is given by
  $$\begin{aligned} f(r)=c_1\frac{e^{-\sqrt{\frac{\delta _1}{\delta _2}}r}}{r}+c_2 \frac{e^{\sqrt{\frac{\delta _1}{\delta _2}}r}}{\sqrt{\delta _1/\delta _2}r}-\frac{\delta _2}{\delta _1}\frac{1}{r}. \end{aligned}$$
  As in the one-dimensional case both limits $\delta _1\rightarrow 0$ and $\delta _2\rightarrow 0$ do not exist.
- Suppose that $n=4$ and that $\frac{\delta _1}{\delta _2}>0$. Then the solution of (3.2) is given by
  $$\begin{aligned} f(r)=&\,c_1\frac{J_1\left( \sqrt{\frac{\delta _1}{\delta _2}}r\right) }{r} +c_2\frac{Y_1\left( \sqrt{\frac{\delta _1}{\delta _2}}r\right) }{r} \\&+\frac{\pi }{2\sqrt{\delta _1/\delta _2}r}\left( J_1\left( \sqrt{\frac{\delta _1}{\delta _2}}r\right) Y_0\left( \sqrt{\frac{\delta _1}{\delta _2}}r\right) -J_0\left( \sqrt{\frac{\delta _1}{\delta _2}}r\right) Y_1\left( \sqrt{\frac{\delta _1}{\delta _2}}r\right) \right) . \end{aligned}$$
  If $\frac{\delta _1}{\delta _2}<0$, then we obtain the solution by an analytic continuation.
- The qualitative behavior of solutions to (3.2) for $n\ge 5$ seems to be the same as above.

It becomes obvious that solutions of (3.1) may show a different qualitative behavior compared to biharmonic functions. Moreover, as one should expect, the qualitative behavior depends heavily on the sign of the product $\delta _1\delta _2$.

### Interpolating Sesqui-Harmonic Curves on the Three-Dimensional Sphere

In this subsection we study interpolating sesqui-harmonic curves on three-dimensional spheres with the round metric, where we follow the ideas from [7].

To this end let $(N,g)$ be a three-dimensional Riemannian manifold with constant sectional curvature $K$. Moreover, let $\gamma :I\rightarrow N$ be a smooth curve that is parametrized by arc length. Let $\{T,N,B\}$ be an orthonormal frame field of $TN$ along the curve $\gamma$. Here, $T=\gamma '$ is the unit tangent vector of $\gamma$, $N$ the unit normal field, and $B$ is chosen such that $\{T,N,B\}$ forms a positive oriented basis.

In this setup we have the following Frenet equations for the curve $\gamma$

3.3 $$\begin{aligned} \nabla _TT=k_gN,\qquad \nabla _TN=-k_gT+\tau _gB,\qquad \nabla _TB=-\tau _gN. \end{aligned}$$

#### Lemma 3.3

Let $\gamma :I\rightarrow N$ be a curve in a three-dimensional Riemannian manifold. Then the curve $\gamma$ is interpolating sesqui-harmonic if the following equation holds

$$\begin{aligned} (-3\delta _2k_gk_g')T+(\delta _2(k_g''-k_g^3-k_g\tau _g^2+k_gK)-\delta _1k_g)N +\delta _2(2k_g'\tau _g+k_g\tau '_g)B=0. \end{aligned}$$

#### Proof

Making use of the Frenet equations (3.3) a direct calculation yields

$$\begin{aligned} \nabla ^3_TT=(-3k_gk_g')T+(k_g''-k_g^3-k_g\tau ^2_g)N+(2k_g'\tau _g+k_g\tau _g')B=0. \end{aligned}$$

Using that the sectional curvature of $N$ is given by $K=R^N(T,N,N,T)$, we obtain the claim. $\square$

#### Corollary 3.4

Let $\gamma :I\rightarrow N$ be a curve in a three-dimensional Riemannian manifold. Then the curve $\gamma$ is interpolating sesqui-harmonic if the following system holds

$$\begin{aligned} k_gk_g'=0,\qquad 2k_g'\tau _g+k_g\tau '_g=0,\qquad \delta _2(k_g''-k_g^3-k_g\tau _g^2+k_gK)=\delta _1k_g. \end{aligned}$$

The non-geodesic solutions $(k_g\ne 0)$ of this system are given by

3.4 $$\begin{aligned} k_g=const\ne 0,\qquad \tau _g=const,\qquad \delta _2(k_g^2+\tau _g^2)=\delta _2K-\delta _1. \end{aligned}$$

We directly obtain the following characterization of interpolating sesqui-harmonic curves:

#### Proposition 3.5

1. Let $\gamma :I\rightarrow N$ be a curve in a three-dimensional Riemannian manifold. If $K\le \frac{\delta _1}{\delta _2}$, then any interpolating sesqui-harmonic curve is a geodesic.
2. To obtain a non-geodesic interpolating sesqui-harmonic curve $\gamma :I\rightarrow S^3$, we have to demand that $\delta _2>\delta _1$.

#### Proposition 3.6

Let $\gamma :I\rightarrow S^3$ be a curve on the three-dimensional sphere with the round metric. The curve $\gamma$ is interpolating sesqui-harmonic if the following equation holds

3.5 $$\begin{aligned} \gamma ^{''''}+(1-\delta _1+\delta _2)\gamma ^{''}+(-k_g^2-\delta _1+\delta _2)\gamma =0. \end{aligned}$$

#### Proof

Differentiating the first equation of (3.3) we find

$$\begin{aligned} \nabla ^2_TN=&-k_g'T-k_g\nabla _TT+\tau '_gB+\tau _g\nabla _TB \\ =&-k_g\nabla _TT+\tau _g\nabla _TB \\ =&-(k_g^2+\tau _g^2)N \\ =&(-\delta _2+\delta _1)N, \end{aligned}$$

where we used (3.4). Moreover, employing the formula for the Levi-Civita connection on $S^3\subset \mathbb {R}^4$

$$\begin{aligned} \nabla _TX=X'+\langle T,X\rangle \gamma , \end{aligned}$$

we get the equations

$$\begin{aligned} \nabla ^2_TN=&\nabla _T(N'+\langle T,N\rangle \gamma ) =N''+\langle T,\nabla _TN\rangle \gamma =\,N''-k_g\gamma , \\ \nabla _TT=&k_gN=\gamma ''+|\gamma '|^2\gamma =\gamma ''+\gamma . \end{aligned}$$

Combining the equations for $\nabla ^2_TN$ we obtain

$$\begin{aligned} N''-k_g\gamma =(\delta _1-\delta _2)N \end{aligned}$$

and rewriting this as an equation for $\gamma$ yields the claim. $\square$

#### Proposition 3.7

Let $\gamma :I\rightarrow S^3$ be a curve on the three-dimensional sphere with the round metric. If $k_g^2=\delta _2-\delta _1$, the interpolating sesqui-harmonic curves are given by

3.6 $$\begin{aligned} \gamma (t)=\left( \frac{\cos \left( \sqrt{(1-\delta _1+\delta _2)}t\right) }{\sqrt{(1-\delta _1 +\delta _2)}},\frac{\sin \left( \sqrt{(1-\delta _1+\delta _2)}t\right) }{\sqrt{(1-\delta _1 +\delta _2)}},d_1,d_2\right) , \end{aligned}$$

where $$\frac{1}{1-\delta _1+\delta _2}+d_1^2+d_2^2=1$$.

#### Proof

Making use of the assumptions (3.5) simplifies as

$$\begin{aligned} \gamma ^{''''}+(1-\delta _1+\delta _2)\gamma ^{''}=0. \end{aligned}$$

Solving this differential equation together with the constraints $|\gamma |^2=1$ and $|\gamma '|^2=1$ yields the claim. $\square$

#### Remark 3.8

Note that it is required in (3.6) that $1-\delta _1+\delta _2>1$, which is equivalent to $\delta _2>\delta _1$. This is consistent with the assumption $k_g^2=\delta _2-\delta _1$.

#### Theorem 3.9

Let $\gamma :I\rightarrow S^3$ be a curve on the three-dimensional sphere with the round metric and suppose that $\delta _2>\delta _1$. Then the non-geodesic solution to (3.5) is given by

3.7 $$\begin{aligned} \gamma (t)&=\frac{1}{\sqrt{a_1^2-a_2^2}}\left( \sqrt{1-a_2^2}\cos (a_1t), \sqrt{1-a_2^2}\sin (a_1t),\sqrt{a_1^2-1}\cos (a_2t),\right. \nonumber \\&\qquad \left. \sqrt{a_1^2-1}\sin (a_2t)\right) \end{aligned}$$

with the constants

$$\begin{aligned} a_1&:=\frac{1}{\sqrt{2}}\sqrt{1-\delta _1+\delta _2+\sqrt{(1+\delta _1 -\delta _2)^2+4k_g^2}}, \\ a_2&:=\frac{1}{\sqrt{2}}\sqrt{1-\delta _1+\delta _2-\sqrt{(1+\delta _1-\delta _2) ^2+4k_g^2}}. \end{aligned}$$

#### Proof

The most general ansatz for a solution of (3.5) is given by

$$\begin{aligned} \gamma (t)=c_1\cos (at)+c_2\sin (at)+c_3\cos (bt)+c_4\sin (bt), \end{aligned}$$

where $c_i,i=1\ldots 4$ are mutually perpendicular and $a$ and $b$ are real numbers. This leads to the following quadratic equation for both $a$ and $b$

$$\begin{aligned} a^4-a^2(1-\delta _1+\delta _2)+(-k_g^2-\delta _1+\delta _2)=0. \end{aligned}$$

We obtain the two solutions

$$\begin{aligned} a_1^2&=\frac{1}{2}\left( 1-\delta _1+\delta _2+\sqrt{(1+\delta _1 -\delta _2)^2+4k_g^2}\right) , \\ a_2^2&=\frac{1}{2}\left( 1-\delta _1+\delta _2-\sqrt{(1+\delta _1-\delta _2)^2+4k_g^2}\right) . \end{aligned}$$

Moreover, the constraints $|\gamma |^2=1$ and $|\gamma '|^2=1$ give the two equations

$$\begin{aligned} |c_1|^2+|c_2|^2=1,\qquad a_1^2|c_1|^2+a_2^2|c_2|^2=1. \end{aligned}$$

Solving this system for $|c_1|^2$ and $|c_2|^2$ yields the claim. $\square$

#### Remark 3.10

If we analyze the constants appearing in (3.7), then we find that we have to demand the condition $\delta _2-\delta _1>k_g^2>0$ in order to obtain a real-valued constant $a_2$. In addition, we find

$$\begin{aligned} a_1^2-a_2^2&=\sqrt{(1+\delta _1-\delta _2)^2+4k_g^2}>0, \\ 1-a_2^2&=\frac{1}{2}(1+\delta _1-\delta _2+\sqrt{(1+\delta _1-\delta _2)^2+4k_g^2})>0,\\ a_1^2-1&=\frac{1}{2}(-1-\delta _1+\delta _2+\sqrt{(1+\delta _1-\delta _2)^2+4k_g^2})>0 \end{aligned}$$

such that we do not get any further restrictions. We conclude that we get a solution of (3.4) for all $\delta _1,\delta _2$ satisfying $\delta _2-\delta _1>k_g^2>0$.

#### Remark 3.11

If we compare our results with [7, Theorem 3.3], then we find that interpolating sesqui-harmonic curves on $S^3$ have the same qualitative behavior as biharmonic curves. More precisely, we have the following two cases:

1. If $k_g^2=\delta _2-\delta _1$, then $\gamma$ is a circle of radius $\frac{1}{\sqrt{1+k_g^2}}$.
2. If $\delta _2-\delta _1>k_g^2>0$, then $\gamma$ is a geodesic of the rescaled Clifford torus
  $$\begin{aligned}&S^1\left( \frac{\sqrt{(1+\delta _1-\delta _2+\sqrt{(1+\delta _1-\delta _2)^2 +4k_g^2})}}{\sqrt{2}((1+\delta _1-\delta _2)^2+4k_g^2)^\frac{1}{4}}\right) \\&\quad \times S^1\left( \frac{\sqrt{-1-\delta _1+\delta _2+\sqrt{(1+\delta _1-\delta _2)^2 +4k_g^2}}}{\sqrt{2}((1+\delta _1-\delta _2)^2+4k_g^2)^\frac{1}{4}}\right) . \end{aligned}$$

Note that the solutions from above reduce to biharmonic curves (see [7, Theorem 3.3]) in the case of $\delta _1=0,\delta _2=1$.

We want to close this subsection by mentioning that it is possible to generalize the results obtained from above to higher-dimensional spheres as was done for biharmonic curves in [8].

## The Qualitative Behavior of Solutions

In this section we study the qualitative behavior of interpolating sesqui-harmonic maps.

In the case of a one-dimensional domain and the target being a Riemannian manifold, the Euler–Lagrange equation reduces to

4.1 $$\begin{aligned} \delta _2\nabla ^3_{\gamma '}\gamma '=\delta _2R^N(\gamma ',\nabla _{\gamma '}\gamma ') \gamma '+\delta _1\nabla _{\gamma '}\gamma ', \end{aligned}$$

where $\gamma :I\rightarrow N$ and $\gamma '$ denotes the derivative with respect to the curve parameter $s$.

### Proposition 4.1

Suppose that $\gamma :I\rightarrow N$ is a smooth solution of (1.4). Then the following conservation type law holds

$$\begin{aligned} \left( \delta _2\frac{\mathrm{d}^3}{\mathrm{d}s^3}-\delta _1\frac{\mathrm{{d}}}{\mathrm{{d}}s}\right) \frac{1}{2}|\gamma '|^2= \delta _2\frac{\mathrm{{d}}}{\mathrm{{d}}s}\frac{3}{2}|\nabla _{\gamma '}\gamma '|^2. \end{aligned}$$

### Proof

We test (4.1) with $\gamma '$ and obtain

$$\begin{aligned} \delta _2\langle \nabla ^3_{\gamma '}\gamma ',\gamma '\rangle =\delta _1\langle \nabla _{\gamma '}\gamma ',\gamma '\rangle =\frac{1}{2}\delta _1\frac{\mathrm{{d}}}{\mathrm{{d}}s}|\gamma '|^2. \end{aligned}$$

The left-hand side can be further simplified as

$$\begin{aligned} \langle \nabla ^3_{\gamma '}\gamma ',\gamma '\rangle =&\frac{\mathrm{{d}}}{\mathrm{{d}}s}\langle \nabla ^2_{\gamma '}\gamma ',\gamma '\rangle -\langle \nabla ^2_{\gamma '}\gamma ', \nabla _{\gamma '}\gamma '\rangle \\ =&\frac{\mathrm{d}^2}{\mathrm{d}s^2}\langle \nabla _{\gamma '}\gamma ',\gamma '\rangle -\frac{3}{2} \frac{\mathrm{{d}}}{\mathrm{{d}}s}|\nabla _{\gamma '}\gamma '|^2 \\ =&\frac{\mathrm{d}^3}{\mathrm{d}s^3}\frac{1}{2}\langle \gamma ',\gamma '\rangle -\frac{3}{2} \frac{\mathrm{{d}}}{\mathrm{{d}}s}|\nabla _{\gamma '}\gamma '|^2, \end{aligned}$$

which completes the proof. $\square$

As already stated in the introduction, it is obvious that harmonic maps solve (1.4). We will give several conditions under which interpolating sesqui-harmonic maps must be harmonic generalizing several results from [19, 27]. To achieve these results we will frequently make use of the following Bochner formula:

### Lemma 4.2

Let $\phi :M\rightarrow N$ be a smooth solution of (1.4). Then the following Bochner formula holds

4.2 $$\begin{aligned} \Delta \frac{1}{2}|\tau (\phi )|^2=|\nabla \tau (\phi )|^2+\langle R^N(\mathrm{{d}}\phi (e_\alpha ),\tau (\phi ))\mathrm{{d}}\phi (e_\alpha ),\tau (\phi )\rangle + \frac{\delta _1}{\delta _2}|\tau (\phi )|^2. \end{aligned}$$

### Proof

This follows by a direct calculation. $\square$

### Proposition 4.3

Suppose that $(M,h)$ is a compact Riemannian manifold. Let $\phi :M\rightarrow N$ be a smooth solution of (1.4).

1. If $N$ has non-positive curvature $K^N\le 0$ and $\delta _1,\delta _2$ have the same sign, then $\phi$ is harmonic.
2. If $|\mathrm{{d}}\phi |^2\le \frac{\delta _1}{|R^N|_{L^\infty }\delta _2}$ and $\delta _1,\delta _2$ have the same sign, then $\phi$ is harmonic.

### Proof

The first statement follows directly from (4.2) by application of the maximum principle. For the second statement we estimate (4.2) as

$$\begin{aligned} \Delta \frac{1}{2}|\tau (\phi )|^2=|\nabla \tau (\phi )|^2+\left( \frac{\delta _1}{\delta _2}-|R^N|_{L^\infty }|\mathrm{{d}}\phi |^2\right) |\tau (\phi )|^2\ge 0 \end{aligned}$$

due to the assumptions. The claim follows again due to the maximum principle. $\square$

If we do not require $M$ to be compact, we can give the following result.

### Proposition 4.4

Let $\phi :M\rightarrow N$ be a Riemannian immersion that solves (1.4) with $|\tau (\phi )|=const$. If $N$ has non-positive curvature $K^N\le 0$ and $\delta _1,\delta _2$ have the same sign, then $\phi$ must be harmonic.

### Proof

Via the maximum principle we obtain $\nabla \tau (\phi )=0$ from (4.2). By assumption the map $\phi$ is an immersion such that

$$\begin{aligned} -|\tau (\phi )|^2=\langle \mathrm{{d}}\phi ,\nabla \tau (\phi )\rangle \end{aligned}$$

concluding the proof. $\square$

In the case that $\dim M=\dim N-1$ the assumption of $N$ having negative sectional curvature can be replaced by demanding negative Ricci curvature.

### Theorem 4.5

Let $\phi :M\rightarrow N$ be a Riemannian immersion. Suppose that $M$ is compact and $\dim M=\dim N-1$. If $N$ has non-positive Ricci curvature and $\delta _1,\delta _2$ have the same sign, then $\phi$ is interpolating sesqui-harmonic if and only if it is harmonic.

### Proof

Since $\phi$ is an immersion and $\dim M=\dim N-1$, we obtain

$$\begin{aligned} R^N(\mathrm{{d}}\phi (e_\alpha ),\tau (\phi ))\mathrm{{d}}\phi (e_\alpha )=-{\text {Ric}}^N(\tau (\phi )). \end{aligned}$$

Making use of (4.2) we get

$$\begin{aligned} \Delta \frac{1}{2}|\tau (\phi )|^2=|\nabla \tau (\phi )|^2-\langle {\text {Ric}}^N(\tau (\phi )),\tau (\phi )\rangle +\frac{\delta _1}{\delta _2}|\tau (\phi )|^2\ge 0 \end{aligned}$$

due to the assumptions. The result follows by the maximum principle. $\square$

As for harmonic maps ([29, Theorem 2]) we can prove a unique continuation theorem for interpolating sesqui-harmonic maps. To obtain this result we recall the following ([1, p.248]):

### Theorem 4.6

Let $A$ be a linear elliptic second-order differential operator defined on a domain $D$ of $\mathbb {R}^n$. Let $u=(u^1,\ldots ,u^n)$ be functions in $D$ satisfying the inequality

4.3 $$\begin{aligned} |Au^j|\le C\left( \sum _{\alpha ,i}\left| \frac{\partial u^i}{\partial x^\alpha }\right| +\sum _i|u^i|\right) . \end{aligned}$$

If $u=0$ in an open set, then $u=0$ throughout $D$.

Making use of this result we can prove the following:

### Proposition 4.7

Let $\phi \in C^4(M,N)$ be an interpolating sesqui-harmonic map. If $\phi$ is harmonic on a connected open set $W$ of $M$, then it is harmonic on the whole connected component of $M$ which contains $W$.

### Proof

The analytic structure of the interpolating sesqui-harmonic map equation is the following:

$$\begin{aligned} |\Delta \tau (\phi )|\le C(|\mathrm{{d}}\phi |^2|\tau (\phi )|+|\tau (\phi )|). \end{aligned}$$

In order to apply Theorem 4.6 we consider the equation for interpolating sesqui-harmonic maps in a coordinate chart in the target. The bound on $|\mathrm{{d}}\phi |^2$ can be obtained by shrinking the chart if necessary such that (4.3) holds.

### Interpolating Sesqui-Harmonic Maps with Vanishing Energy-Momentum Tensor

In this section we study the qualitative behavior of solutions to (1.4) under the additional assumption that the energy-momentum tensor (2.2) vanishes similar to [22]. Such an assumption is partially motivated from physics: In physics one usually also varies the action functional (1.3) with respect to the metric on the domain and the resulting Euler–Lagrange equation yields the vanishing of the energy-momentum tensor.

In the following we will often make use of the trace of the energy-momentum tensor (2.2), which is given by (where $n=\dim M$)

4.4 $$\begin{aligned} {\text {Tr}}T=\delta _1\left( 1-\frac{n}{2}\right) |\mathrm{{d}}\phi |^2+\delta _2\frac{n}{2}|\tau (\phi )|^2 +\delta _2(n-2)\langle \mathrm{{d}}\phi ,\nabla \tau (\phi )\rangle . \end{aligned}$$

Note that we do not have to assume that $\phi$ is a solution of (1.4) in the following.

#### Proposition 4.8

Let $\gamma :S^1\rightarrow N$ be a curve with vanishing energy-momentum tensor. If $\delta _1\delta _2>0$, then $\gamma$ maps to a point.

#### Proof

Using (4.4) and integrating over $S^1$ we find

$$\begin{aligned} 0=\frac{\delta _1}{2}\int _{S^1}|\gamma '|^2\mathrm{d}s+\frac{3}{2}\delta _2\int _{S^1}| \tau (\gamma )|^2\mathrm{d}s, \end{aligned}$$

which yields the claim. $\square$

#### Proposition 4.9

Suppose that $(M,h)$ is a Riemannian surface. Let $\phi :M\rightarrow N$ be a smooth map with vanishing energy-momentum tensor. Then $\phi$ is harmonic.

#### Proof

Since $\dim M=2$, we obtain from (4.4) that $|\tau (\phi )|^2=0$ yielding the claim. $\square$

For a higher-dimensional domain we have the following result.

#### Proposition 4.10

Let $\phi :M\rightarrow N$ be a smooth map with vanishing energy-momentum tensor. Then the following statements hold:

1. If $\dim M=3$ and $\delta _1\delta _2<0$, then $\phi$ is trivial.
2. If $\dim M=4$, then $\phi$ is trivial.
3. If $\dim M\ge 5$ and $\delta _1\delta _2>0$, then $\phi$ is trivial.

#### Proof

Integrating (4.4) we obtain

$$\begin{aligned} 0=\delta _1\left( 1-\frac{n}{2}\right) \int _M|\mathrm{{d}}\phi |^2\text {d}V+\delta _2\left( 2-\frac{n}{2}\right) \int |\tau (\phi )|^2\text {d}V, \end{aligned}$$

which already yields the result. $\square$

As a next step we rewrite the condition on the vanishing of the energy-momentum tensor.

#### Proposition 4.11

Let $\phi :M\rightarrow N$ be a smooth map and assume that $n\ne 2$. Then the vanishing of the energy-momentum tensor is equivalent to

4.5 $$\begin{aligned} 0&=T(X,Y)=\delta _1\langle \mathrm{{d}}\phi (X),\mathrm{{d}}\phi (Y)\rangle \nonumber \\&\quad -\delta _2\frac{1}{n-2}|\tau (\phi )|^2h(X,Y) -\delta _2\big (\langle \mathrm{{d}}\phi (X),\nabla _Y\tau (\phi )\rangle +\langle \mathrm{d}\phi (Y),\nabla _X\tau (\phi )\rangle \big ).\quad \end{aligned}$$

#### Proof

Rewriting the equation for the vanishing of the trace of the energy-momentum tensor (4.4), we find

$$\begin{aligned} \delta _2\langle \mathrm{{d}}\phi ,\nabla \tau (\phi )\rangle =\delta _1\frac{(\frac{n}{2}-1)}{(n-2)} |\mathrm{{d}}\phi |^2-\delta _2\frac{n}{2(n-2)}|\tau (\phi )|^2.\quad \end{aligned}$$

Inserting this into the energy-momentum tensor (2.2) yields the claim. $\square$

This allows us to give the following:

#### Proposition 4.12

Let $\phi :M\rightarrow N$ be a smooth map with vanishing energy-momentum tensor. Suppose that $\dim M>2$ and ${\text {rank}}\,\phi \le n-1$. Then $\phi$ is harmonic.

#### Proof

Fix a point $p\in M$. By assumption ${\text {rank}}\,\phi \le n-1$ and hence there exists a vector $X_p\in \ker \mathrm{{d}}\phi _p$. For $X=Y=X_p$ we can infer from (4.5) that $\tau (\phi )=0$ yielding the claim. $\square$

If the domain manifold $M$ is non-compact we can give the following variant of the previous results.

#### Proposition 4.13

Let $\phi :M\rightarrow N$ be a smooth Riemannian immersion with vanishing energy-momentum tensor. If $\dim M=2$, then $\phi$ is harmonic, and if $\dim M=4$, then $\phi$ is trivial.

#### Proof

Since $\phi$ is a Riemannian immersion, we have $\langle \tau (\phi ),\mathrm{{d}}\phi \rangle =0$. Hence (4.4) yields

$$\begin{aligned} 0=\delta _1\left( 1-\frac{n}{2}\right) |\mathrm{{d}}\phi |^2+\delta _2\left( 2-\frac{n}{2}\right) |\tau (\phi )|^2, \end{aligned}$$

which proves the claim.

### Conformal Construction of Interpolating Sesqui-Harmonic Maps

In [3] the authors present a powerful construction method for biharmonic maps. Instead of trying to directly solve the fourth-order equation for biharmonic maps they assume the existence of a harmonic map and then perform a conformal transformation of the metric on the domain to render this map biharmonic. In particular, they call a metric that renders the identity map biharmonic, a *biharmonic metric*. In this section we will discuss if the same approach can also be used to construct interpolating sesqui-harmonic maps.

To this end let $\phi :(M,h)\rightarrow (N,g)$ be a smooth map. If we perform a conformal transformation of the metric on the domain, that is $\tilde{h}=e^{2u}h$ for some smooth function $u$, we have the following formula for the transformation of the tension field

$$\begin{aligned} \tau _{\tilde{h}}(\phi )=e^{-2u}(\tau _h(\phi )+(n-2)\mathrm{{d}}\phi (\nabla u)), \end{aligned}$$

where $\tau _{\tilde{h}}(\phi )$ denotes the tension field of the map $\phi$ with respect to the metric $\tilde{h}$. In addition, we set $n:=\dim M$.

Now, suppose that $\phi$ is a harmonic map with respect to $h$, that is $\tau _h(\phi )=0$, then we obtain the identity

$$\begin{aligned} \tau _{\tilde{h}}(\phi )=e^{-2u}(n-2)\mathrm{{d}}\phi (\nabla u). \end{aligned}$$

This allows us to deduce

#### Proposition 4.14

Let $\phi :(M,h)\rightarrow (N,g)$ be a smooth harmonic map and suppose that $\dim M\ne 2$. Let $\tilde{h}=e^{2u}h$ be a metric conformal to $h$. Then the map $\phi :(M,\tilde{h})\rightarrow (N,g)$ is interpolating sesqui-harmonic if and only if

$$\begin{aligned} \delta _2\big (\nabla ^*\nabla \mathrm{{d}}\phi (\nabla u)+(n-6)\nabla _{\nabla u}\mathrm{{d}}\phi (\nabla u) +2(-\Delta u-(n-4)|\mathrm{{d}}u|^2)\mathrm{{d}}\phi (\nabla u) \\ \nonumber +{\text {Tr}}_hR^N(\mathrm{{d}}\phi (\nabla u),\mathrm{{d}}\phi )\mathrm{{d}}\phi \big ) =\delta _1e^{-2u}\mathrm{{d}}\phi (\nabla u). \end{aligned}$$

#### Proof

For every $v\in \Gamma (\phi ^*TN)$ the following formula holds

$$\begin{aligned} \nabla ^*_{\tilde{h}}\nabla _{\tilde{h}}v=e^{-2u}(\nabla ^*_{h} \nabla _{h}v+(n-2)\nabla _{\nabla u}v). \end{aligned}$$

By a direct calculation we find

$$\begin{aligned} \nabla _h^*\nabla _h(\tau _{\tilde{h}}(\phi )) =&(n-2)e^{-2u}\big (-2\Delta u\mathrm{{d}}\phi (\nabla u) +4|\mathrm{{d}}u|^2\mathrm{{d}}\phi (\nabla u) \\&-4\langle \nabla u,\nabla (\mathrm{{d}}\phi (\nabla u))\rangle +\nabla ^*\nabla \mathrm{{d}}\phi (\nabla u)\big ). \end{aligned}$$

Together with

$$\begin{aligned} \nabla _{\nabla u}\tau _{\tilde{h}}(\phi )&=(n-2)e^{-2u}(-2|\nabla u|^2\mathrm{{d}}\phi (\nabla u)+\nabla _{\nabla u}\mathrm{{d}}\phi (\nabla u)), \\ R^N(\mathrm{{d}}\phi ,\tau _{\tilde{h}}(\phi ))\mathrm{{d}}\phi&=(n-2)e^{-4u}R^N(\mathrm{{d}}\phi ,\mathrm{d}\phi (\nabla u))\mathrm{{d}}\phi , \end{aligned}$$

this completes the proof. $\square$

In the following we will call a metric that renders the identity map interpolating sesqui-harmonic an *interpolating sesqui-harmonic metric*.

#### Corollary 4.15

Let $\phi :(M,h)\rightarrow (M,h)$ be the identity map and suppose that $\dim M\ne 2$. Let $\tilde{h}=e^{2u}h$ be a metric conformal to $h$. Then the map $\phi :(M,\tilde{h})\rightarrow (M,h)$ is interpolating sesqui-harmonic if and only if

$$\begin{aligned}&\delta _2(2(-\Delta u-(n-4)|\mathrm{{d}}u|^2)\nabla u +(n-6)\nabla _{\nabla u}\nabla u+{\text {Tr}}_h(\nabla ^*\nabla )\nabla u +{\text {Ric}}^M(\nabla u))\\&\quad =\delta _1e^{-2u}\nabla u. \end{aligned}$$

We now rewrite this as an equation for $\nabla u$.

#### Proposition 4.16

Let $\phi :(M,h)\rightarrow (M,h)$ be the identity map and suppose that $\dim M\ne 2$. Let $\tilde{h}=e^{2u}h$ be a metric conformal to $h$ and set $\nabla u=\beta$. Then the map $\phi :(M,\tilde{h})\rightarrow (M,h)$ is interpolating sesqui-harmonic if and only if

4.6 $$\begin{aligned} -\Delta \beta =2(\mathrm{{d}}^*\beta -(n-4)|\beta |^2)\beta +(n-6)\frac{1}{2}\mathrm{{d}}|\beta |^2 +2{\text {Ric}}^M(\beta ^\sharp )^\flat -\frac{\delta _1}{\delta _2}e^{-2u}\beta , \end{aligned}$$

where $-\Delta =\mathrm{{dd}}^*+\mathrm{{d}}^*\mathrm{{d}}$ is the Laplacian acting on one-forms.

#### Proof

The Laplacian acting on one-forms satisfies the following Weitzenböck identity:

$$\begin{aligned} \Delta \beta (X)={\text {Tr}}_h(\nabla ^2)\beta (X)-\beta {\text {Ric}}(X), \end{aligned}$$

where $X$ is a vector field. In addition, note that (see [3, Proof of Proposition 2.2] for more details)

$$\begin{aligned} \nabla _{\nabla u}\nabla u=\frac{1}{2}\mathrm{{d}}|\beta |^2, \end{aligned}$$

which completes the proof. $\square$

#### Proposition 4.17

Let $(M,h)$ be a compact manifold of strictly negative Ricci curvature with $\dim M>2$ and assume that $\delta _1\delta _2>0$. Then there does not exist an interpolating sesqui-harmonic metric that is conformally related to $h$ except a constant multiple of $h$.

#### Proof

Note that our sign convention for the Laplacian is different from the one used in [3]. We define the one-form $\theta :=e^{-2u}\beta$. By a direct calculation we then find using (4.6)

$$\begin{aligned} \Delta \theta =-\frac{1}{2}(n-2)e^{2u}\mathrm{{d}}|\theta |^2-2{\text {Ric}}^M (\theta ^\sharp )^\flat +\frac{\delta _1}{\delta _2}e^{-2u}\theta . \end{aligned}$$

In addition, we have

$$\begin{aligned} \Delta \frac{1}{2}|\theta |^2&=\langle \Delta \theta ,\theta \rangle +|\nabla \theta |^2+{\text {Ric}}(\theta ^\sharp ,\theta ^\sharp ) \\&=-\frac{1}{2}(n-2)e^{2u}\langle \mathrm{{d}}|\theta |^2,\theta \rangle +|\nabla \theta |^2-{\text {Ric}} (\theta ^\sharp ,\theta ^\sharp )+\frac{\delta _1}{\delta _2}e^{-2u}|\theta |^2. \end{aligned}$$

The claim then follows by the maximum principle.

#### Remark 4.18

In contrast to the case of biharmonic maps (4.6) contains also a term involving $u$ on the right-hand side. This reflects the fact that both harmonic and biharmonic maps on its own have a nice behavior under conformal deformations of the domain metric, whereas interpolating sesqui-harmonic maps do not. This prevents us from making a connection between interpolating sesqui-harmonic metrics and isoparametric functions as was done in [3] for biharmonic maps.

### A Liouville-Type Theorem for Interpolating Sesqui-Harmonic Maps Between Complete Manifolds

In this section we will prove a Liouville-type theorem for solutions of (1.4) between complete Riemannian manifolds generalizing a similar result for biharmonic maps from [25]. For more Liouville-type theorems for biharmonic maps see [5, 6] and references therein.

To this end we will make use of the following result due to Gaffney [14]:

#### Theorem 4.19

Let $(M,h)$ be a complete Riemannian manifold. If a $C^1$ one-form $\omega$ satisfies

$$\begin{aligned} \int _M|\omega |{\mathrm{{d}}V}<\infty \qquad \text { and }\qquad \int _M|\delta \omega |{\mathrm{{d}}V}<\infty \end{aligned}$$

or, equivalently, a $C^1$ vector field $X$ defined by $\omega (Y)=h(X,Y)$, satisfies

$$\begin{aligned} \int _M|X|{\mathrm{{d}}V}<\infty \qquad \text { and }\qquad \int _M{\text {div}}(X){\mathrm{{d}}V}<\infty , \end{aligned}$$

then

$$\begin{aligned} \int _M(\delta \omega )\mathrm{{d}}V=\int _M{\text {div}}(X){\mathrm{{d}}V}=0. \end{aligned}$$

#### Theorem 4.20

Let $(M,h)$ be a complete non-compact Riemannian manifold and $(N,g)$ a manifold with non-positive sectional curvature. Let $\phi :M\rightarrow N$ be a smooth solution of (1.4) and $p$ be a real constant satisfying $2\le p<\infty$.

1. If $$\delta _1\delta _2>0$$ and
  $$\begin{aligned} \int _M|\tau (\phi )|^p{\mathrm{{d}}V}<\infty ,\qquad \int _M|\mathrm{{d}}\phi |^2{\mathrm{{d}}V}<\infty , \end{aligned}$$
  then $\phi$ must be harmonic.
2. If $$\delta _1\delta _2>0$$, $${{\text {vol}}}(M,h)=\infty $$ and
  $$\begin{aligned} \int _M|\tau (\phi )|^p{\mathrm{{d}}V}<\infty , \end{aligned}$$
  then $\phi$ must be harmonic.

#### Proof

We choose a cutoff function $0\le \eta \le 1$ on $M$ that satisfies

$$\begin{aligned} \eta (x)=1\text { for } x\in B_R(x_0),\qquad \eta (x)=0\text { for } x\in B_{2R}(x_0),\qquad |\nabla \eta |\le \frac{C}{R}\text { for } x\in M, \end{aligned}$$

where $B_R(x_0)$ denotes the geodesic ball around the point $x_0$ with radius $R$.

We test the interpolating sesqui-harmonic map equation (1.4) with $\eta ^2\tau (\phi )|\tau (\phi )|^{p-2}$ and find

$$\begin{aligned}&\eta ^2|\tau (\phi )|^{p-2}\langle \Delta \tau (\phi ),\tau (\phi )\rangle = \eta ^2|\tau (\phi )|^{p-2}\langle R^N(\mathrm{{d}}\phi (e_\alpha ),\tau (\phi ))\mathrm{{d}}\phi (e_\alpha ),\tau (\phi )\rangle \\&\quad +\frac{\delta _1}{\delta _2}\eta ^2|\tau (\phi )|^{p} \ge 0, \end{aligned}$$

where we made use of the assumptions on the curvature of the target and the signs of $\delta _1$ and $\delta _2$. Integrating over $M$ and using integration by parts we obtain

$$\begin{aligned}&\int _M\eta ^2|\tau (\phi )|^{p-2}\langle \Delta \tau (\phi ),\tau (\phi ) \rangle \text {d}V\\&\quad =-2\int _M\langle \nabla \tau (\phi ),\tau (\phi )\rangle |\tau (\phi )|^{p-2} \eta \nabla \eta \text {d}V\\&\qquad -(p-2)\int _M\eta ^2|\langle \nabla \tau (\phi ),\tau (\phi )\rangle |^2| \tau (\phi )|^{p-4}\text {d}V\\&\qquad -\int _M\eta ^2|\nabla \tau (\phi )|^2|\tau (\phi )|^{p-2}\text {d}V\\&\quad \le \frac{C}{R^2}\int _M|\tau (\phi )|^{p}\text {d}V-\frac{1}{2}\int _M\eta ^2|\nabla \tau (\phi )|^2|\tau (\phi )|^{p-2}\text {d}V\\&\qquad -(p-2)\int _M\eta ^2|\langle \nabla \tau (\phi ),\tau (\phi )\rangle |^2|\tau (\phi )|^{p-4}\text {d}V, \end{aligned}$$

where we used Young’s inequality and the properties of the cutoff function $\eta$. Combining both equations we find

$$\begin{aligned}&\frac{1}{2}\int _M\eta ^2|\nabla \tau (\phi )|^2|\tau (\phi )|^{p-2}\text {d}V\le \frac{C}{R^2}\int _M|\tau (\phi )|^{p}\text {d}V\\&\quad -(p-2)\int _M\eta ^2|\langle \nabla \tau (\phi ),\tau (\phi )\rangle |^2|\tau (\phi )|^{p}\text {d}V. \end{aligned}$$

Letting $R\rightarrow \infty$ and using the finiteness assumption of the $L^p$ norm of the tension field, we may deduce that $\tau (\phi )$ is parallel and thus has constant norm.

To establish the first claim of the theorem, we make use of Theorem 4.19. We define a one-form $\omega$ by

$$\begin{aligned} \omega (X):=|\tau (\phi )|^{\frac{p}{2}-1}\langle \mathrm{{d}}\phi (X),\tau (\phi )\rangle , \end{aligned}$$

where $X$ is a vector field on $M$. Note that

$$\begin{aligned} \int _M|\omega |\text {d}V\le \int _M|\mathrm{{d}}\phi ||\tau (\phi )|^{\frac{p}{2}}\text {d}V\le \Big (\int _M|\mathrm{{d}}\phi |^2\text {d}V\Big )^\frac{1}{2}\Big (\int _M|\tau (\phi )|^p\text {d}V\Big )^\frac{1}{2}<\infty . \end{aligned}$$

Using that $|\tau (\phi )|$ has constant norm we find by a direct calculation that $\delta \omega =|\tau (\phi )|^{\frac{p}{2}+1}$. Again, since $|\tau (\phi )|$ has constant norm and the $L^p$-norm of $\tau (\phi )$ is bounded, we find that $|\delta \omega |$ is integrable over $M$. By application of Theorem 4.19 we can then deduce that $\tau (\phi )=0$.

To prove the second claim, we note that ${{\text {vol}}}(M,h)=\infty$ and $|\tau (\phi )|\ne 0$ give

$$\begin{aligned} \int _M|\tau (\phi )|^p\text {d}V=|\tau (\phi )|^p{{\text {vol}}}(M,h)=\infty , \end{aligned}$$

yielding a contradiction.

## Footnotes

1 Finding an appropriate name for solutions of (1.4) turned out to be subtle. The author would like to thank John Wood for suggesting the word “sesqui.”
